# Case Report: A *MiT* family translocation renal cell carcinoma in the renal pelvis, calyces and upper ureter misdiagnosed as upper tract urothelial carcinoma

**DOI:** 10.3389/fonc.2023.1197578

**Published:** 2023-08-16

**Authors:** Yuhua Zou, Linwei Liu, Xiaojuan Xie, Cunzhi Zhong, Qinlin Wang, Sheng Yan, Quanliang Liu

**Affiliations:** ^1^ Department of Urology, The First Affiliated Hospital of Gannan Medical University, Ganzhou, China; ^2^ Department of Cardiology, The First Affiliated Hospital of Gannan Medical University, Ganzhou, China; ^3^ Department of Anesthesiology, The First Affiliated Hospital of Gannan Medical University, Ganzhou, China

**Keywords:** renal cell carcinoma, upper tract urothelial carcinoma, renal pelvis, upper ureter, MiT family translocation RCC, misdiagnosed, ureteroscopy

## Abstract

**Background:**

Upper tract urothelial carcinoma (UTUC) is the most common urothelial malignancy in the renal pelvis or ureter. Renal pelvic carcinoma accounts for 90% of all tumours in the renal pelvis, so the mass in the renal pelvis is usually considered a UTUC. Renal cell carcinoma (RCC) in the renal pelvis, calyces and upper ureter is extremely rare, especially *MiT* family translocation RCC, which makes this case even more uncommon.

**Case presentation:**

We report the case of a 54-year-old man had intermittent painless gross haematuria with occasional blood clots and urodynia for 2 years. Contrast-enhanced computed tomography (CT) and magnetic resonance imaging (MRI) scan showed an enlarged left kidney, and a soft tissue mass was seen in the renal pelvis, calyces and upper ureter. The patient’s urine-based cytology was positive three times. Due to the severity of the upper ureteral lumen stenosis, we did not perform pathological biopsy during ureteroscopy. In the current case, clinical symptoms, imaging examinations, urine-based cytology, and ureteroscopy were combined to obtain a preoperative diagnosis of UTUC. Therefore, robot-assisted laparoscopic left radical nephroureterectomy and retroperitoneal lymphadenectomy were performed. Unexpectedly, the patient was pathologically diagnosed with *MiT* family translocation RCC after surgery. The surgery was uneventful. There was no intestinal tube injury or other complications perioperatively. The postoperative follow-up was satisfactory.

**Conclusion:**

*MiT* family translocation RCC in the renal pelvis, calyces and upper ureter is extremely rare, and can be easily confused with UTUC, resulting in the expansion of surgical scope. Preoperative ureteroscopy and biopsy or tumour punch biopsy should be used to obtain accurate pathology as far as possible, and the selection of correct surgical method is conducive to a good prognosis for patients.

## 1 Introduction

Renal cell carcinoma (RCC) is a common urinary tract malignancy, accounting for 2%-3% of adult malignancies and 80%-85% of all renal tumours (1). RCC originates from the epithelial cells of the renal tubules, whereas upper tract urothelial carcinoma (UTUC) originates from the renal pelvis or the ureter (2, 3). The different origins and biologies of the two types of tumours necessitate different surgical treatments. Occasionally, RCC invades the renal pelvis and calyces, but RCC in the renal pelvis, calyces and upper ureter is extremely rare. In the current report, typical clinical symptoms, imaging of a mass in the renal pelvis, calyces and upper ureter, and positive urine-based cytology resulted in a preoperative diagnosis of UTUC. Robot-assisted laparoscopic left radical nephroureterectomy and retroperitoneal lymphadenectomy were performed. The postoperative pathological diagnosis was *MiT* family translocation RCC. In this case report, a *MiT* family translocation RCC was located in the renal pelvis, calyces and upper ureter, which was misdiagnosed preoperatively as UTUC. The findings of this case are summarized below.

## 2 Case presentation

**Figure 1:**
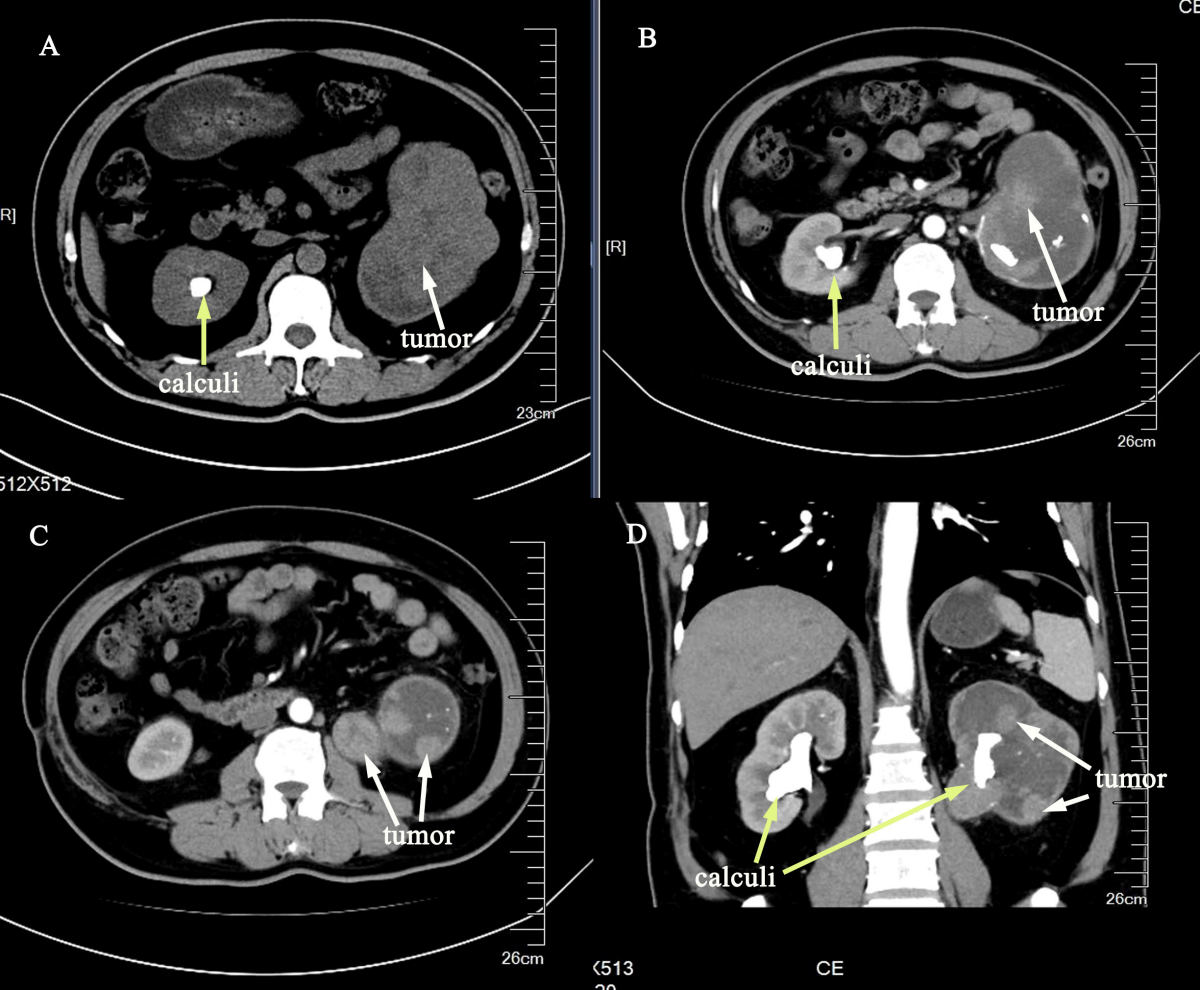
Contrast-enhanced CT scan with an irregular soft tissue mass shadow in the left renal pelvis, calyces and upper ureter **(A–D)**, the mass was significantly enhanced in the arterial stage **(B–D)**. Multiple nodular dense shadows were observed in both kidneys **(A–D)**. (The tumor was showed by white arrows, and the calculi was showed by yellow arrows.).

**Figure 2:**
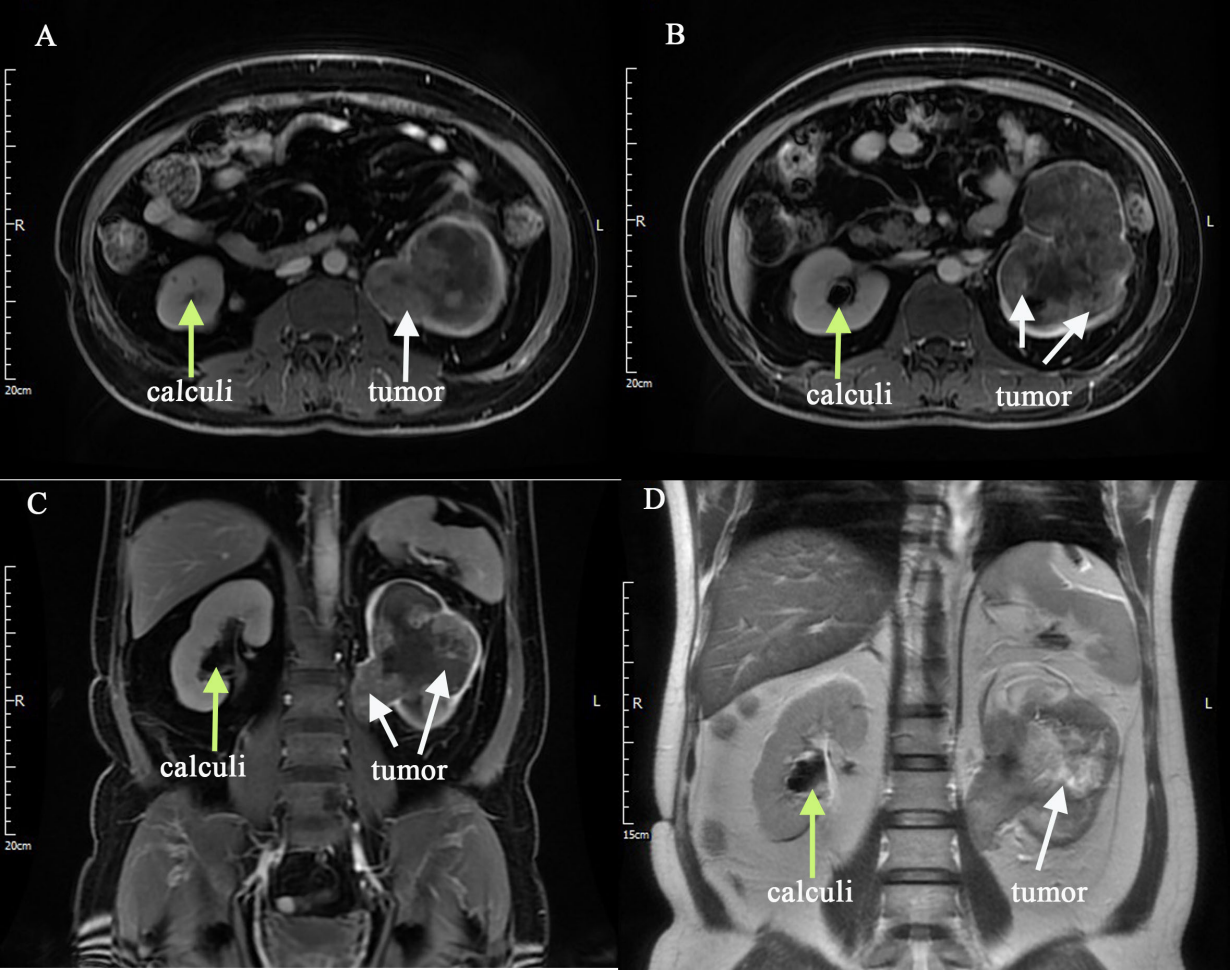
Contrast-enhanced MRI scan was performed **(A–D)**, and it showed an enlarged left kidney, and a soft tissue mass was seen in the renal pelvis, calyces and upper ureter, and with bilateral renal calculi. (The tumor was showed by white arrows, and the calculi was showed by yellow arrows).

The reporting of this study conforms to CARE guidelines (4). Written informed consent was obtained from the patient for the publication of this case report. A 54-year-old man had intermittent painless gross haematuria with occasional blood clots and urodynia for 2 years. The symptoms of haematuria were relieved by oral antibiotics and proper rest. The patient underwent contrast-enhanced computed tomography (CT) scan during a health check-up, which revealed bilateral renal calculi, a soft tissue mass in the left renal pelvis, calyces and upper ureter, therefore, a tumorous lesion was suspected (**Figures 1A–D**). The patient had a history of smoking for more than 10 years. He also had a 12-year history of hypertension, and his blood pressure was well controlled using regular oral antihypertensive drugs. Regarding his previous surgical history, the patient underwent thymectomy in 2002, open pyelotomy for right renal pelvis stone in 2004, and surgical treatment for a left lower limb tibial fracture due to trauma in 2019, with no history of special medication or diabetes. Concerning preoperative biochemical examination, blood analysis showed haemoglobin 88 g/L and normal liver and kidney function. Contrast-enhanced magnetic resonance imaging (MRI) scan showed an enlarged left kidney, and a soft tissue mass was seen in the renal pelvis, calyces and upper ureter, with an area of 10.8 × 7.5 cm. T1WI showed a mixed signal, T2WI showed a mixed, slightly high signal, and DWI showed an inhomogeneous high signal (**Figures 2A–D**). The enhancement scan showed mild to moderate inhomogeneous enhancement in the arterial phase, and the degree of enhancement in the venous and delayed phases was reduced. The renal parenchyma was compressed and thinned. A nodular T2WI low signal was observed in the bilateral calyces(**Figures 2A–D**). Retroperitoneal lymph nodes were seen. The bladder was well filled, the bladder wall was not thick, the signal intensity was uniform, and no nodules or masses were seen. Chest CT did not show any significant abnormality. The estimated glomerular filtration rates (eGFR) were 8.48 and 38.56 ml/min for the left and right kidneys, respectively. All three urine-based cytology examinations were positive. The abnormal nucleo-cytoplasm ratio were seen microscopically and considered tumour cells. To further clarify the diagnosis, left ureteroscopy and cystoscopy were performed under combined spinal-epidural anaesthesia. Intraoperatively, a tortuous upper ureter on the left side was noted with an uneven mucosal surface and narrow lumen, which prevented the scope from going up to the renal pelvis, and no pathological biopsy was taken. No abnormal manifestations were seen in the bladder. The patient was preoperatively diagnosed with left-sided UTUC with renal calculus and hydronephrosis based on the typical clinical presentation, imaging features of UTUC, positive urine-based cytology, and ureteroscopic findings.

**Figure 3:**
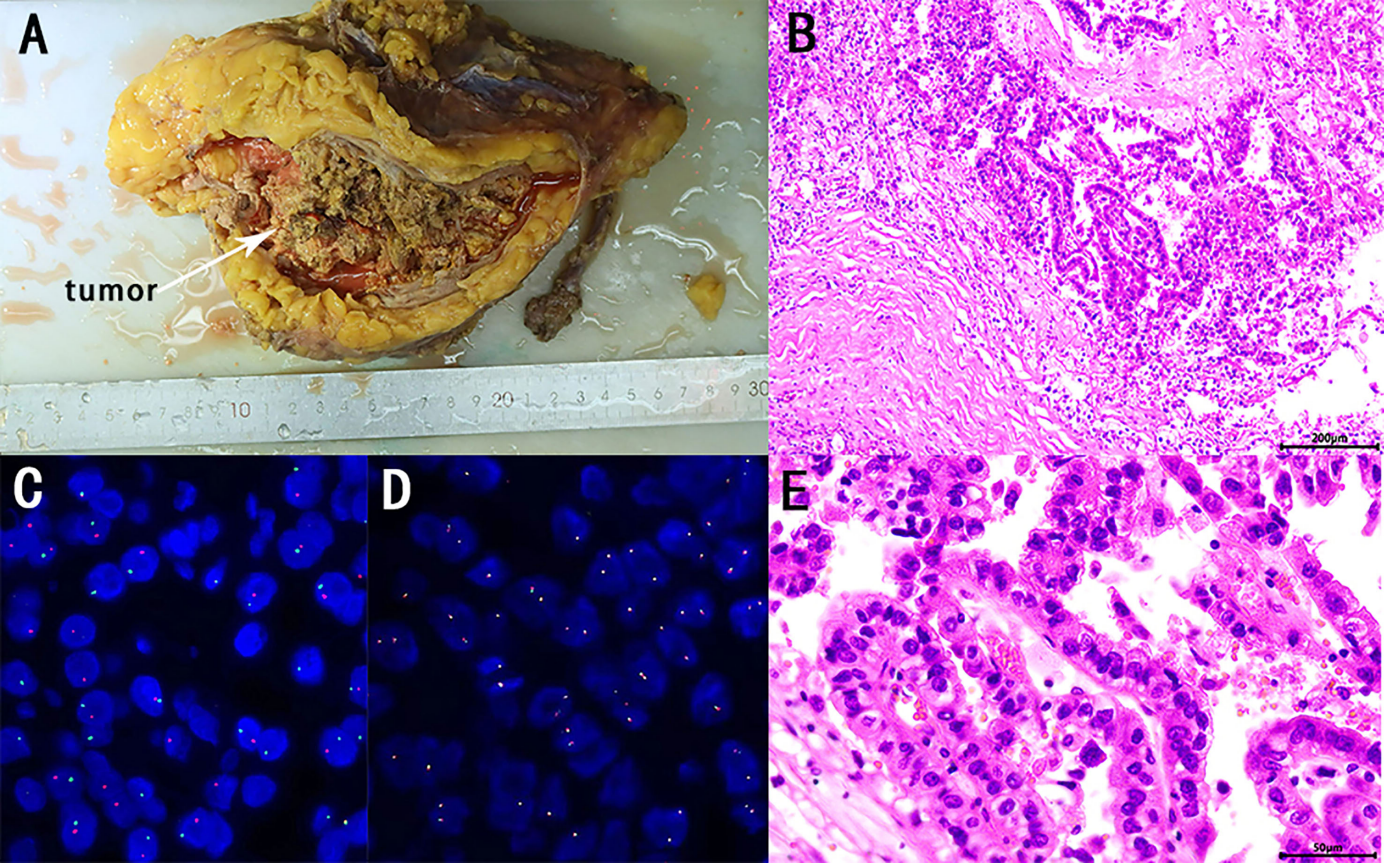
Macroscopic view of the tumor (white arrow) **(A)**. Microscopic overview of the tumor **(B, E)**. [Hematoxylin-eosin staining, ×100 **(B)**, ×400 **(E)**] *TFE3* and *TFEB* fluorescence *in situ* hybridization (FISH) assay on paraffin embedded tissue. *TFE3* and *TFEB* gene Break Apart Probes, confirmed the *TFE3* gene rearrangement in tumor cells (one red and one green signals) **(C)**, and no *TFEB* gene break rearrangement detected **(D)**.

**Figure 4:**
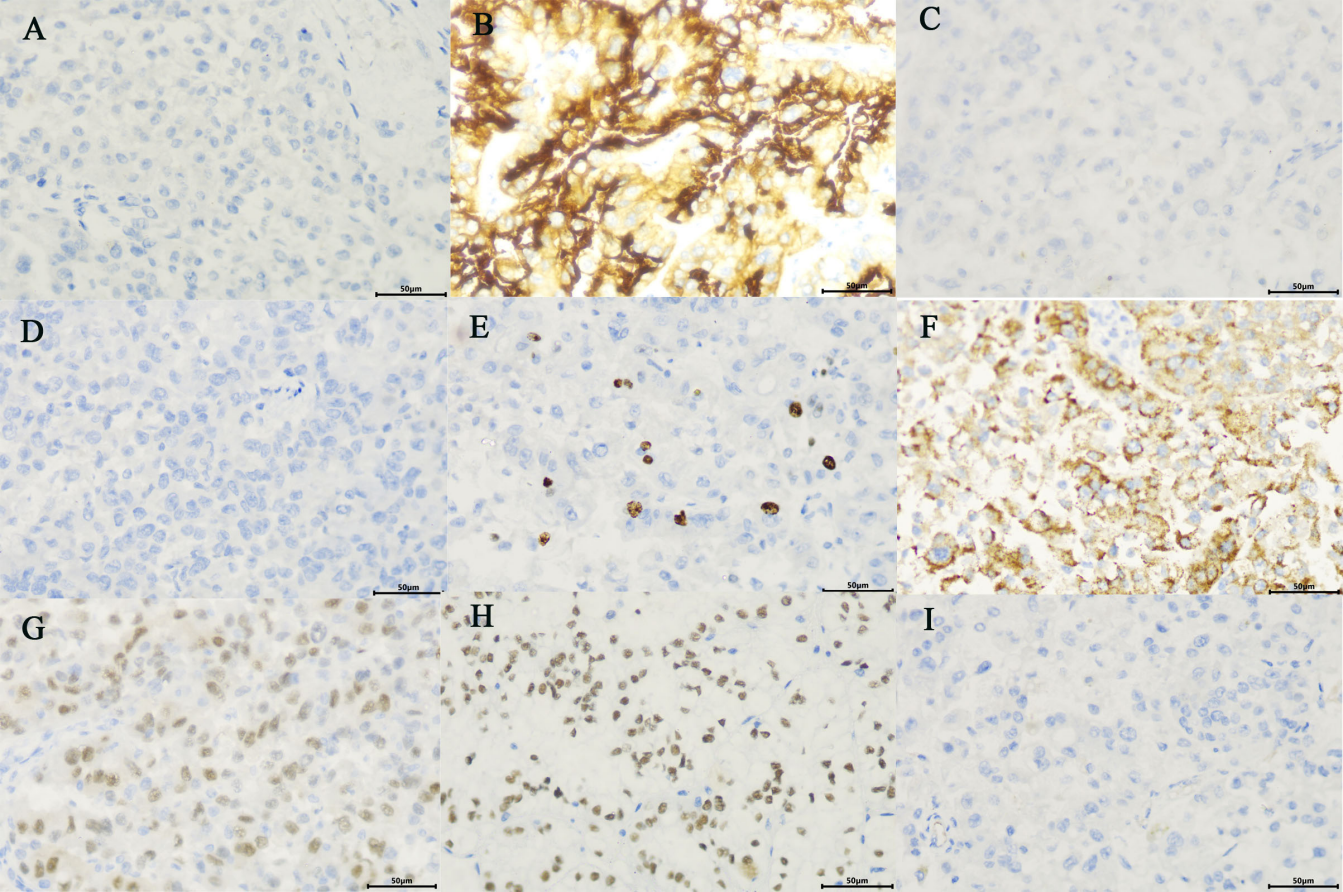
Immunohistochemistrical (IHC) analysis **(A–I)**: **(A)** CAIX(−), **(B)** CD10 (+), **(C)** CK7(−), **(D)** CK20 (−), **(E)** Ki-67 (about 5%+), **(F)**.AMACR (+), **(G)** PAX8 (+), **(H)** TFE3 (+), **(I)** vimentin (−), considered to be *MiT* family translocation RCC (in the left upper urinary tract). (×400).

The patient and his family consented to surgery. Robot-assisted laparoscopic left radical nephroureterectomy and retroperitoneal lymphadenectomy were performed under general anaesthesia on October 9. The operation was uneventful and lasted 280 min, with a blood loss of 400 ml, and 2 U of filtered white suspended red blood cells were infused intraoperatively. The specimen was removed from the body, and the kidney and ureter seemed intact. The specimen was excised, and a soft tissue mass was seen from the upper pole of the kidney to the renal pelvis and upper ureter, with a size of about 10 × 7 cm, clearly demarcated from the surrounding tissues (**Figure 3A**). Hematoxylin-eosin (HE) staining and pathology considered as RCC, to be further confirmed by immunohistochemical examination (**Figures 3B, E**). The tumour was adjacent to the peritoneum, and no renal pelvis invasion was noted. No definite choroidal aneurysm embolus or nerve bundle invasion was seen. No cancer was seen in the ureter, ureteral and vascular cut edge, retroperitoneal lymph nodes were detected, and no cancer metastasis was seen (0/15). Immunohistochemical results showed that CAIX (−), CD10 (+), CK7 (−), CK20(−), Ki-67 (about 5%+),AMACR (+), PAX8 (+), TFE3 (+) and vimentin (−), indicated *MiT* family translocation RCC (in the left upper urinary tract) with massive necrosis (**Figures 4A–I**). Considering the previous findings and the immunohistochemical examination, *MiT* family translocation RCC was suspected, and performing molecular testing was suggested to further confirm the diagnosis. Subsequently, a fluorescence *in situ* hybridization (FISH) study, using *TFE3* and *TFEB* gene Break Apart Probes, confirmed the *TFE3* gene rearrangement in tumor cells, with the break-apart signal was 58.5% (**Figure 3C**), and no *TFEB* gene break rearrangement detected (**Figure 3D**). The postoperative pathological stage was pT2bN0M0, stage I. There was no intestinal tube injury or other complications perioperatively. The patient was satisfied with the treatment. The patient was discharged from the hospital 8 days after surgery, and was followed up regularly for 4 months with no secondary infection, intestinal obstruction or local recurrence.

## 3 Discussion

UTUC is the most common urothelial malignancy in the renal pelvis or ureter. It accounts for 5 to 10% of all urothelial carcinomas in the European and American populations (2, 3). The prevalent clinical symptom is often associated with gross hematuria or microscopic hematuria (5). Currently, the diagnosis of UTUC is mainly based on imaging, urine-based cytology, ureteroscopy and biopsy. Among imaging examinations, computed tomography urography (CTU) and magnetic resonance urography (MRU) have high accuracy for UTUC diagnosis (6). In particular, with a sensitivity of 92% and specificity of 95%, CTU can be the first choice for diagnosing and staging UTUC (7). Although the patient’s urine-based cytology was positive three times, there was still a possibility of false positives. Because the patient has upper urinary tract stones, the abnormal nucleo-cytoplasm ratio of the shed cells may also be caused by long-term chronic inflammatory stimulation caused by the stones. However, if conditions permit, a preoperative combination of FISH, immunocytology and tumor marker nuclear matrix protein 22 (NMP22) with good sensitivity and specificity may increase diagnostic accuracy (8, 9).

In this case, the patient had bilateral kidney calculi. Most researchers believe that kidney stones can increase the risk of RCC and UTUC, especially in patients under 40 years who present with kidney stones for the first time. However, the increased risk of UTUC is not associated with stones in the renal pelvis or ureter (10). The increased risk is usually due to chronic inflammatory irritation and infection caused by stones (11), as well as the potentially harmful solutes in the urine. All these may lead to changes in urothelial proliferation and play a carcinogenic role. Moreover, stones form in the proximal tubules, and tumor development may be associated with stone-forming salts in the proximal tubular filtrate. The presence of these solutes may affect cellular metabolism, which may lead to an increased risk of papillary RCC (10).

Obtaining an accurate pathologic diagnosis of UTUC before surgery is currently a clinical challenge. However, histopathological evidence obtained using ureteroscopy is the gold standard for diagnosis. Studies have shown some variation in matching ureteroscopy and biopsy results to the final pathologic tumor grading: 66% for low grade and 97% for high grade (12). However, several studies have shown that preoperative ureteroscopy is more advantageous for detecting small lesions and obtaining direct exfoliative cell sampling from the renal pelvis or ureter. Certainly, there is a greatly increased risk of postoperative tumor bladder recurrence (13), but ureteroscopy and biopsy can still confirm the diagnosis in most patients. In the current case, a tortuous upper ureter on the left side with an uneven mucosal surface and tortuous narrow lumen was found via ureteroscopy. UTUC was considered due to the patient’s clinical symptoms of painless gross hematuria, the soft tissue mass in the renal pelvis, calyces and upper ureter on MRI and CT, and three positive urine-based cytology examinations. Based on this consideration and the severity of the upper ureteral lumen stenosis, we did not perform pathological biopsy during ureteroscopy. Unfortunately, this did not allow a direct pathologic histologic basis for the diagnosis in this case.

Renal pelvic carcinoma accounts for 90% of all tumors in the renal pelvis, so the mass in the renal pelvis is usually considered a UTUC. Although renal cancer can invade and penetrate the renal pelvis and calyces, RCC in the renal pelvis, calyces and upper ureter is rare, and only a few cases are reported clinically (12, 14–18). Therefore, it is vital to note that when imaging suggests a tumor in the renal pelvis and calyces, it does not always indicate UTUC, RCC or metastasis should also be considered (19, 20). The pathogenesis for RCC located in the renal pelvis, calyces, and upper ureter remains unclear. Previous reports suggest the following possible mechanisms. First, because of the hollow structure of the renal pelvis, it is more vulnerable than the parenchyma when limited RCC originates in the marginal parenchyma surrounding the renal pelvis or when RCC invades the entire kidney (12, 14). Second, tumor cells rapidly proliferate through implantation or invasion of the urinary tract mucosa and subsequently in the lumen, invading the ureter or even down to the bladder (15, 18, 20). In this case, postoperative pathology confirmed that RCC did not invade the renal pelvis and upper urethral mucosa, but a mass was formed in the renal pelvis, calyces and upper ureter, which further led to severe stenosis of the upper ureter. Therefore, staging RCC in the renal pelvis, calyces and upper ureter according to the current TNM staging system also poses new problems and challenges (14, 21, 22).

The correct preoperative diagnosis of masses in the renal pelvis and calyces can help choose a treatment plan. UTUC and RCC have different origins and biological features, requiring different surgical treatments. Some reports showed that laparoscopic radical ureterectomy performed by an experienced operator and following tumor-free principles is equivalent to open radical ureterectomy in terms of perioperative safety and tumor control (23, 24). In the current case, robot-assisted laparoscopic left radical nephroureterectomy and retroperitoneal lymphadenectomy were performed. Unexpectedly, the patient was pathologically diagnosed with *MiT* family translocation RCC after surgery.

*MiT* family translocation RCC is a rare RCC subtype. It was introduced in the 2016 WHO Classification of Tumors of the Urinary and Renal System (25), and it involves fusion genes including *MiT/TFE* family genes, *MITF*, *TFEB* and *TFE3* (26). It is highly malignant, accounting for approximately 1%–5% of incidental RCC cases in adults (27). The clinical presentations of *MiT* family translocation RCC are nonspecific, including painless gross hematuria and lower back pain similar to UTUC. The main body of the lesion is mostly within the renal medulla, with solid and cystic structures, and its image shows common manifestations such as hemorrhage, necrosis, cystic degeneration, calcification and pseudo-envelope. The solid component is slightly hyperintense on CT, slightly high-signal on MRI T1WI, slightly low-signal on MRI T2WI, and high-signal on MRI DWI (28, 29). Surgical resection is currently the treatment of choice, mainly including radical nephrectomy (RN) and partial nephrectomy (PN), which can be selected according to the patient’s age, tumor size, and the presence of lymph nodes or distant metastases (29, 30).

The patient in our report had an uneventful surgery and good postoperative recovery, achieving the same therapeutic outcome without other side effects. However, due to the preoperative misdiagnosis of UTUC, an unnecessary full-length ureteral and bladder sleeve resection was still performed to ensure the best tumor control outcome. Moreover, an ipsilateral retroperitoneal lymphadenectomy was performed, which expanded the scope of surgery and caused more trauma, a lesson worth reflecting on. For tumor cases with suspicious preoperative diagnosis, intraoperative frozen pathology examination can be used to improve treatment accuracy and determine subsequent treatment methods to avoid more trauma. However, for patients with suspected UTUC, intraoperative frozen pathology will not only destroy the integrity of the ureter and undermine the tumor-free principle but also easily lead to abdominal implantation of the tumor, so it was not performed in this case. Because of the severe ureteral stenosis noted via ureteroscopy in this patient, biopsy specimens could not be obtained. The patient might have benefited more if a further preoperative punch biopsy of the tumor had been performed, which is worthy of further exploration.

## 4 Conclusions

In conclusion, *MiT* family translocation RCC in the renal pelvis, calyces and upper ureter has been rarely reported and can be easily confused with UTUC. For this reason, RCC or metastasis should also be considered for tumour lesions in the renal pelvis, calyces and collecting system. Before the selection of suspicious cases and surgical procedures, reliable histological evidence should be obtained. Ureteroscopy and biopsy or tumour punch biopsy is necessary, and should be combined with various imaging techniques or FISH. Thus, the preoperative diagnosis can be further confirmed to select the correct treatment method and achieve desirable outcomes.

## Data availability statement

The original contributions presented in the study are included in the article/**Supplementary Material**. Further inquiries can be directed to the corresponding author.

## Ethics statement

Written informed consent was obtained from the participant/patient(s) for the publication of this case report.

## Author contributions

YZ and LL prepared and wrote the article. LL was directly involved in the management of the patients. XX, CZ and QW were responsible for the collection and organization of the literature. QL revised the manuscript and acted as corresponding authors. All authors contributed to the article and approved the submitted version.
