# Supplementary material for: Case Report: A MiT family translocation renal cell carcinoma in the renal pelvis, calyces and upper ureter misdiagnosed as upper tract urothelial carcinoma
Source: Front Oncol. 2023 Aug 16;13:1197578. doi: 10.3389/fonc.2023.1197578 (PMC10468567; doi:10.3389/fonc.2023.1197578)
Supplement: Supplementary file 1 [file DataSheet_1.pdf]

CARE Checklist of information to include when writing a case report

CARE case report guidelines

CC BY-NC-ND

Topic	Item	Checklist item description	Reported on Line
Title	1	The diagnosis or intervention of primary focus followed by the words "case report"	Page1/Line1–2
Key Words	2	2 to 5 key words that identify diagnoses or interventions in this case report, including "case report"	Page1/Line15-16
Abstract (no references)	3a	Introduction: What is unique about this case and what does it add to the scientific literature?	Page2/Line42-45
	3b	Main symptoms and/or important clinical findings	Page2/Line51-58
	3c	The main diagnoses, therapeutic interventions, and outcomes	Page2-3/Line82-104
	3d	Conclusion—What is the main "take-away" lesson(s) from this case?	Page5/Line190-196
Introduction	4	One or two paragraphs summarizing why this case is unique (**may include references**)	Page4/Line140-163
Patient Information	5a	De-identified patient specific information	Page2/Line51-52
	5b	Primary concerns and symptoms of the patient	Page2/Line52-55
	5c	Medical, family, and psycho-social history including relevant genetic information	Page2/Line58-62
	5d	Relevant past interventions with outcomes	Page2/Line58-59
Clinical Findings	6	Describe significant physical examination (PE) and important clinical findings	Page2/Line62-74
Timeline	7	Historical and current information from this episode of care organized as a timeline	Page2/Line58-62
Diagnostic Assessment	8a	Diagnostic testing (such as PE, laboratory testing, imaging, surveys).	Page2/Line55-82
	8b	Diagnostic challenges (such as access to testing, financial, or cultural)	Page4/Line140-143
	8c	Diagnosis (including other diagnoses considered)	Page2/Line81-84
	8d	Prognosis (such as staging in oncology) where applicable	Page3/Line101-104
Therapeutic Intervention	9a	Types of therapeutic intervention (such as pharmacologic, surgical, preventive, self-care)	Page3/Line85-87
	9b	Administration of therapeutic intervention (such as dosage, strength, duration)	Page3/Line87-88
	9c	Changes in therapeutic intervention (with rationale)	N/A
Follow-up and Outcomes	10a	Clinician and patient-assessed outcomes (if available)	Page3/Line101-102
	10b	Important follow-up diagnostic and other test results	Page3/Line102-104
	10c	Intervention adherence and tolerability (How was this assessed?)	Page3/Line85-86
	10d	Adverse and unanticipated events	N/A
Discussion	11a	A scientific discussion of the strengths AND limitations associated with this case report	Page5/Line190-196
	11b	Discussion of the relevant medical literature **with references**	Page3-4/Line106-175
	11c	The scientific rationale for any conclusions (including assessment of possible causes)	Page4/Line140-155
	11d	The primary "take-away" lessons of this case report (without references) in a one paragraph conclusion	Page5/Line176-188
Patient Perspective	12	The patient should share their perspective in one to two paragraphs on the treatment(s) they received	Page3/Line102
Informed Consent	13	Did the patient give informed consent? Please provide if requested	Yes ☑ No ☐
